# Disorders of Dental Hard Tissues Induced by Radioiodine-131 (I-131) Therapy Used in Differentiated Thyroid Cancer: An In Vitro Study

**DOI:** 10.3390/biomedicines8110475

**Published:** 2020-11-05

**Authors:** Alexandru Mester, Andra Piciu, Doina Piciu, Ioan Petean, Patricia Ondine Lucaciu, Dragos Apostu, Cristina Moisescu-Goia, Andrada Voina-Tonea, Marioara Moldovan

**Affiliations:** 1 Department of Oral Health, University of Medicine and Pharmacy “Iuliu Hatieganu”, 400012 Cluj-Napoca, Romania; mester.alexandru@umfcluj.ro (A.M.); patricia.lucaciu@umfcluj.ro (P.O.L.); 2 Department of Medical Oncology, University of Medicine and Pharmacy “Iuliu Hatieganu”, 400012 Cluj-Napoca, Romania; 3 Department of Endocrine Tumors and Nuclear Medicine, Oncology Institute “Ion Chiricuta”, University of Medicine and Pharmacy “Iuliu Hatieganu, 400012 Cluj-Napoca, Romania; doina.piciu@umfcluj.ro (D.P.); popcrisro@yahoo.com (C.M-G.); 4 Faculty of Chemistry and Chemical Engineering, University Babes-Bolyai, 400294 Cluj-Napoca, Romania; ipetean@chem.ubbcluj.ro; 5 Department of Orthopedics, University of Medicine and Pharmacy “Iuliu Hatieganu”, 400012 Cluj-Napoca, Romania; Apostu.Dragos@umfcluj.ro; 6 Department of Dental Materials, University of Medicine and Pharmacy “Iuliu Hatieganu”, 400012 Cluj-Napoca, Romania; andrada.tonea@umfcluj.ro; 7 Department of Polymer Composites, Institute of Chemistry “Raluca Ripan”, University Babes-Bolyai, 400294 Cluj-Napoca, Romania; marioara.moldovan@ubbcluj.ro

**Keywords:** oral disease, differentiated thyroid cancer, radioiodine therapy, dental hard tissue

## Abstract

This study aimed to evaluate, in vitro, the effects of I-131 on enamel and dentin in healthy human incisive permanent maxillary teeth. Our in vitro model analogue with the in vivo conditions of differentiated thyroid carcinoma patients treated with I-131, consisted in a solution of I-131 dissolved in artificial saliva. A total of 48 teeth were divided into eight groups (n = 6): control, irradiation groups at 3, 6, 12, 24, 36, 48, and 192 h, respectively. At the end of radiation exposure, radioiodine activity of specimens was assessed. Fine microstructure, nanostructure, surface roughness, and hidroxyapatite (HAP) crystallite diameter were investigated by atomic force microscopy (AFM) to both enamel and dentin structures. There is a constant increase of radioactivity in dental structures at 3, 6, 12, 24 h, due to progressive retention and I-131 migration, with a maximum at 36 h. Enamel showed notable alterations, which was correlated with the increase of the treatment time. A relevant visible distance between the HAP prisms was observed after 24 h. The surface suffered a loss in its compact structure. I-131 acts in the same way on HAP crystallites in dentin as in those in enamel. It was noticed that their morpho-dimensional changes occurred only after 12 h of treatment. Radioiodine-131 determines degradation of enamel and dentin by starting from the alteration of the crystalline network of HAP prisms, transforming them from compact materials into an agglomeration of rocky submicron structures.

## 1. Introduction

Radioiodine therapy with I-131 is the systemic and specific treatment in differentiated thyroid cancer (DTC). Thyroid cancer is the most frequent endocrine cancer, with an important increase of incidence, during the last decades. According to the statistics of the American Cancer Society and National Institute of Health, NIH an estimated 52,890 new cases of thyroid cancer will be diagnosed, in 2020 in the US, representing 2.9% of all new cancer cases, with a 5-year relative survival of 98.3%, representing 0.4% from all cancer deaths [1] and with three in four cases occurring in women [2], surgical indication in case of a thyroid nodule. Due to the high curability of the disease, the quality of life of these patients is a crucial issue, the impact on oral health being one of the important items.

Due to the superficial localization and facile examination, most thyroid cancers are diagnosed at an early stage. Treatment depends on the histology, tumor size, and extent of the disease. The most common histology, more than 85% of cases [1,2,3] is differentiated thyroid carcinoma (papillary and follicular carcinoma), forms that benefit from the systemic and specific therapy with radioiodine (I-131).

I-131 is a beta-emitting radionuclide with a physical half-life of 8.02 days, a principal gamma ray of 364 keV and a principal beta particle with a maximum energy of 0.61 MeV, average energy of 0.192 MeV, and an average range in tissue of 0.4 mm [3].

The activity of administrated radiopharmaceutical is variable, the mean single administered activity ranges from 1.11 to 5.55 GBq; more than 75% of the patients are treated with activities between 1.85 and 2.59 GBq, with a total response in disease free (Tg undetectable in high TSH condition and no anti-Tg) in 89% of cases within 1 year [3].

About 20% of the salivary glands were dysfunctional on salivary gland scintigraphy at 5 years after a single dose ablation, especially when high doses were used; xerostomia was more associated with submandibular gland dysfunction and the prevalence of dysfunctional salivary glands [4,5]. Mandel et al. reported that the salivary glands concentrate iodine 20–100 times more than serum [6]. The principal site of the iodide transport into saliva is the epithelium of the parotid being extracted from periductal capillaries and concentrated by the ductal epithelium, then secreted into the lumen duct and then with saliva in the oral cavity. Some authors [6,7] revealed that up to 24–29% of the administrated activity of I-131 is excreted by saliva into oral cavity. The presence of radioiodine in the saliva after therapy, the physiological uptake in the salivary system led the authors to the hypothesis of possible changes in the dental structure.

Up to this moment, it is known that head and neck radiotherapy has effects on developing rampant decays, mucosal lesions, changes in the quality and flow of the saliva, and microbiota [8,9,10,11]. Another aspect that should be mentioned is that dentin structure is weaker post-radiation and loses its structure to support the enamel [12,13]. Direct radiation was proven to have an impact on the microstructure, crystallinity, and chemical composition of the hard dental tissues [13,14]. According to our research, there is no scientific evidence to support the alteration of mechanic-morphological properties of dental hard tissues after I-131 radiation. For a better understanding, the purpose of this study was to assess in vitro the effects of I-131 on enamel and dentin in permanent maxillary incisive teeth.

## 2. Materials and Methods

### 2.1. Sample Preparation and Distribution in Groups

After approval (IRB no. 17/12.02.2020) of the experimental protocol, 48 healthy human incisive permanent maxillary teeth were collected. All patients included in the study have signed an informed consent and agreed with medical procedure and for using the data in scientific purposes. The teeth were extracted due to periodontal reasons and had the following characteristics: permanent maxillary incisive, no decays, no fillings, no prosthodontic work, no endodontic treatment, or any other defects. To have a confirmation of the smoothness, the specimens collected were washed with water, dried, and examined on microscopic magnification (Olympus SP 350 Digital Camera, Stream Basic Imaging Software, Olympus, Tokyo, Japan). Then, calculus debridement was removed by hand using ultrasonic scaling (Newtron P5 Blend, Bordeaux, Acteon, France) and placement in ultrasonic bath in water for 5 min (Ultrasonic cleaner, Yeson, Ningbo, China). In the end, they were kept in artificial saliva 4 °C for 1 month. The specimens were distributed in groups as follows: control group (CTR, n = 6) and irradiation groups after radioiodine exposure: 3 h (IRD3, n = 6), 6 h (IRD6, n = 6), 12 h (IRD12, n = 6), 24 h (IRD24, n = 6), 36 h (IRD36, n = 6), 48 h (IRD48, n = 6), 192 h (IRD192, n = 6).

### 2.2. Radioiodine Irradiation Basics and Measurements of the Radioactive Isotopes

For thyroid remnant ablation the I-131 activity is ranging between 1.11 (30 mCi) and 2.56 GBq (70 mCi), so the mean I-131 activity is 1.85 GBq (50 mCi) [15,16,17]; an estimated activity of 24-30% is excreted in saliva and into oral cavity [6,7], actually 0.6 GBq (16.6 mCi) of I-131 might be present in the saliva, early after radioiodine therapy. According to Jo KS et al. [7], the whole body scan with I-131 post-therapy performed at 6 days revealed radioiodine retention by 28.9% of parotid glands and similarly of submandibular glands, and no significant differences in the proportions of radioiodine retention were evident among the four glands. Giovanella L. [18] reported that both salivary glands and blood flow increase the radioiodine uptake in 24 h after administration of I-131 therapy, and then reaches a plateau. Taking into account the mentioned data, our in vitro model analogue with the in vivo conditions of DTC patients treated with I-131, consisted in a solution of 16 mCi I-131 dissolved in 50 mL of artificial saliva. In the prepared solution, the specimens were introduced and then, after irradiation, were taken out at 3, 6, 12, 24, 36, 48, and 192 h. The control specimens were immersed in 20 mL of artificial saliva without I-131. To measure the radioactive isotopes, serial measurements of each tooth were done with the calibrator (Curiementor 3, Freiburg, Germany) using the energy and window selection for I-131. Each probe was measured three times and a mean of the values expressed in counts/minutes was calculated.

### 2.3. Atomic Force Microscopy on Enamel and Dentin

At the macroscopic examination all the samples were prepared according to the requirements of the atomic force microscope (AFM) as follows: parallel flat slices with a thickness of 1 mm provided for investigation both enamel and dentin surface. Both enamel and dentin were investigated by AFM (JSPM 4210 microscope, Jeol, Tokyo, Japan). The samples were scanned in tapping mode using cantilevers of the NSC 15 Hard type with a conical type of diamond-coated silicon nitride (Mikromasch Co., Sofia, Bulgaria). The type radius of curvature is 10 nm for optimal image resolution at both the fine microstructural and nanostructural levels. The force constant of the cantilever is 40 N/m and the resonant frequency is 325 kHz. Samples were scanned at different scanning areas starting at 10 × 10 μm up to 1 × 1 μm. The obtained topographic images were processed in standard mode (Win SPM Processing 2.0 processing software, Jeol, Tokyo, Japan). The surface roughness Ra and Rq were measured and the diameter of HAP crystallites (HAP prisms) was measured so as to have a complete characterization of the samples in terms of the evolution of the shape and dimensions of the structural constituents of enamel and dentin as a function of time of exposure to treatment. Centralized analysis of the obtained images showed that the fine microstructure of the samples is optimally highlighted by the images at the area of 5 × 5 μm and the nanostructure by the images at the area of 1 × 1 μm.

### 2.4. Statistical Analysis

All data were analyzed using statistical software (GraphPad Prism 6.0, San Diego, USA). Means, standard deviations (SD), and percentages were calculated, and correlation tests were applied. Normal distribution was checked using Shapiro–Wilk. To compare differences among groups, ANOVA test was used, completed with Bonferroni correction. The results were considered statistically significant when *p* < 0.05.

## 3. Results

### 3.1. Radioiodine Activity 

**Figure 1:**
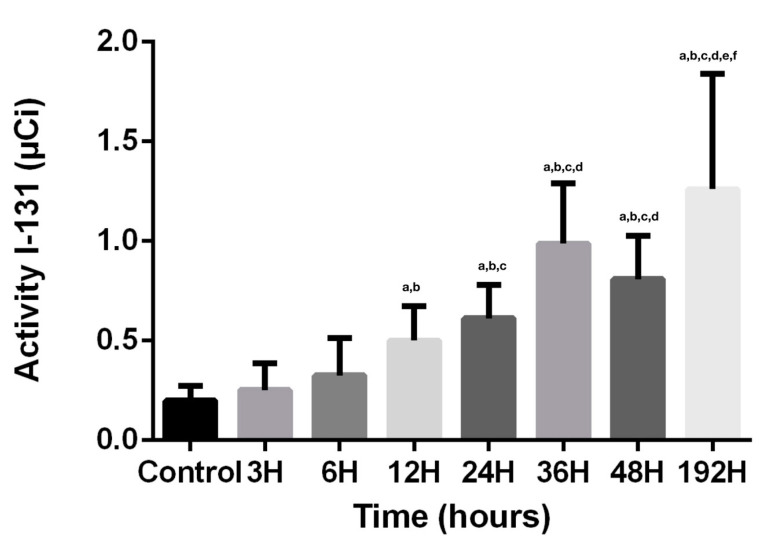
Activity of radioiodine. Values are expressed in mean ± SD. ^a^ *p* < 0.05 compared to CTR group; ^b^ *p* < 0.05 compared to IRD3 group; ^c^ *p* < 0.05 compared to IRD6 group; ^d^ *p* < 0.05 compared to IRD12 group; ^e^ *p* < 0.05 compared to IRD24 group; ^f^ *p* < 0.05 compared to IRD48 group.

Considering that the action of radioiodine on the dental structures, due to its tissue penetrability of maximum 2 mm, is the disruption of crystals and demineralization, the results obtained are strongly concordant with the physiological pathways and physical characteristics. The absorption of radioiodine, demonstrated by direct activity measurements, is directly proportional with the penetrability of radiopharmaceutical agent and time since the first contact (Figure 1). There is a constant increase of activity at 3, 6, 12, 24 h, due to progressive retention and I-131 migration. At 24 h, there is the first step when the physical decay plays a role, because the calculation and indirect measurements needed to be adjusted with the decay factor of 0.918, so the entire activity of the saliva where the teeth were kept decreased by this factor. Despite the decay, the values of the dental activities continued to increase, demonstrating the progress of the penetrability. It is statistically significant (*p* < 0.05) that the maximum penetrance of I-131 in dental structures is at 36 h. The activity measured at 48 h is lowering, having a possible explanation of decay factor, 0.841 and of cessation of penetration and retention of I-131 in dental tissues. A surprising result related to the values of activity in direct measurements was that despite the decay factor of 0.5 at 192 h, these values reached the highest limit, with statistical significance (*p* < 0.05), demonstrating that the process of absorption, penetrance, and retention continued to progress, because of the important damage of the crystals from the dental structures.

### 3.2. Enamel Assessment

**Figure 2:**
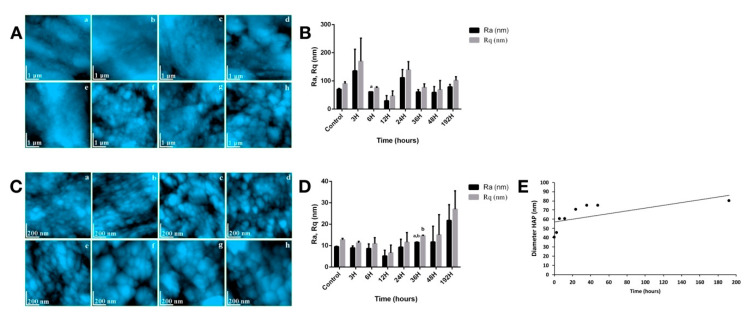
(**A**) Enamel fine microstructure: (a) CTR, (b) 3H, (c) 6H, (d) 12H, (e) 24H, (f) 36H, (g) 48H, (h) 192H; (**B**) roughness surface of enamel fine microstructure; (**C**) enamel nanostructure: (a) CTR, (b) 3H, (c) 6H, (d) 12H, (e) 24H, (f) 36H, (g) 48H, (h) 192H; (**D**) characteristics of enamel nanostructure; (**E**) HAP crystallite diameter. For (**B**,**D**), values are expressed in mean ± SD. ^a^ *p* < 0.05 compared to CTR group; ^b^ *p* < 0.05 compared to IRD3 group.

In Figure 2A, the evolution of the enamel fine microstructure post-radioiodine exposure is presented. CTR group (Figure 2A.a), has a characteristic surface for healthy enamel, with its topography described by a succession of gentle unevenness, related to a very compact enamel with HAP crystallites well highlighted and welded in the structure. After the first 6 h of treatment (Figure 2A.b–d), no major changes in the topography of enamel surface were noticed. The variations of roughness were mainly assigned to the local particularities of the enamel. After 12 h, notable alterations of morphology occurred; represented by local surface depressions uncharacteristically for the typical morphology of healthy enamel. Their progress was correlated with the increase of the treatment time. A relevant visible distance between the HAP prisms was observed after 24 h, the surface suffered a loss in its compact structure, compared to the healthy stage (Figure 2A.e). After 192 h, the enamel surface was strongly altered (Figure 2A.h). Missing HAP crystallites as in case of demineralization and fine Micronics and even submicronic rocky formations (1–1.5 μm diameter and 150–300 nm) that contained HAP crystals, were observed. The drastic change in the surface topography shows that the long penetration of I-131 through the enamel mass caused local demineralization of the structure at a fine microscopic level so as to form “islands” with a rocky appearance. These rock formations contained well-welded HAP crystallites, very difficult to see due to the rugged relief induced by the portions with missing crystallites. The values of the roughness surface of enamel fine microstructure are presented in Figure 2B. The values of the average roughness resulting from the fine microstructure are very dispersed. The only statistical significance (*p* < 0.05) for Ra was reached at 6 h.

At nanostructural level, for healthy enamel (Figure 2C.a), HAP nanoparticles with a round appearance and diameters predominantly around 40 nm were clearly observed (in some places 60 nm). A very high compactness of the surface was observed, resulted from the firm position of the HAP crystallites. The obtained Ra roughness of the surface was in the range of 9.41–18.2 nm, in full accordance to the data in the literature for healthy enamel. The roughness values at nanostructural level are centralized in Figure 2D. The healthy nanostructure appearance of the enamel was maintained after 3 h of treatment. The first changes were visible after 6 h. The shape of HAP became slightly oval and their diameter increased from about 40 nm in the untreated enamel to about 60 nm in the present case (Figure 2C.c). In some areas, demineralization sites were observed, where external crystallites were missing while deeper ones became visible. The effect was progressively accentuated after 12 and 24 h, respectively. From 36 h post-radioiodine, (Figure 2C.f), the morphology of the enamel at the nanostructure level changed drastically. Alteration of HAP crystallites in the enamel surface was observed in form of an eroded structure, with bouldering-looking submicron formations, containing HAP crystallites inside them. The effect of erosion and fragmentation of the enamel cohesion culminated in the treated sample at 192 h post-treatment. The enamel nanostructure showed drastic changes. Instead of well-contoured HAP crystallites with diameters of 40 nm specific to untreated healthy enamel, rocky-rounded formations with the smallest diameters around 80 nm were observed (some over 200 nm). The drastic change in the surface topography at nanostructural level was manifested with a net doubling of the roughness values Ra and Rq (Figure 2D). Statistical significance (*p* < 0.05) was reached for Ra and Rq at 36 h.

Another particularly important parameter was the diameter of HAP crystallites (Figure 2E). Its increase was logarithmic indicating the occurrence of a complex process that does not act linearly on healthy HAP prisms. From a physical point of view, it is difficult for a HAP prism with a diameter of 40 nm to double its diameter. Yet, the AFM analysis showed exactly that.

### 3.3. Dentin Assessment

**Figure 3:**
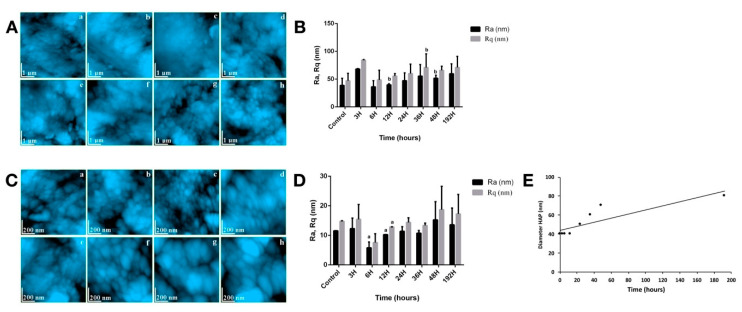
(**A**) Dentin fine microstructure: (a) CTR, (b) 3H, (c) 6H, (d) 12H, (e) 24H, (f) 36H, (g) 48 H, (h) 192H; (**B**) roughness surface of dentin fine microstructure; (**C**) dentin nanostructure: (a) CTR, (b) 3H, (c) 6H, (d) 12H, (e) 24H, (f) 36H, (g) 48 H, (h) 192H; (**D**) characteristics of dentin nanostructure; (**E**) HAP crystallite diameter. For (**B**,**D**), values are expressed in mean ± SD. ^a^ *p* < 0.05 compared to CTR group; ^b^ *p* < 0.05 compared to IRD3 group.

Figure 3A.a captures very well the morphology of the intertubular dentin for a healthy tooth. Relatively parallel well-mineralized strands of HAP nanoparticles with predominantly diameters around 40 nm are observed. The strings are based on the collagen fibers in the dentin structure. The fact that their structure is not detectable in the investigated surface shows a high degree of health of the investigated dentin.

Collagen has a sufficiently strong and at the same time elastic protein network so as to give the dentin a tenacious behavior at different mechanical stresses. Considering the phenomena involved in the alteration of the tooth enamel, in the case of dentin, the presence of the collagenic structure is likely to attenuate the effect of dislocation of the HAP prisms from the structure. This is evidenced by AFM images showing a fine-looking microscopic dentin that is healthy even after 6 h of treatment (Figure 3A.b,c). Significant changes in surface topography occurred after 12 h of treatment (Figure 3A.d), due to the of local bumps with the appearance of depression. These correspond to portions of early demineralization and can be correlated with appropriate changes at the nanostructure level. As the treatment time increases, these dentin alterations progress as seen in Figure 3A.e–g. At 48 h post-radioiodine, the degradation of dentin cohesion is evident by the appearance of fine submicronous boulder structures with diameters in the range of 200–300 nm. Figure 3A.h shows very well the degree of dentin alteration after the 192 h of treatment. It is as if the dentin mass began to crack at the intracrystalline level, rocky formations appearing with dimensions between 200 and 500 nm, which in the interior contain HAP crystallites. These are not detectable due to the very rugged topography of the sample. The Ra and Rq roughness’ were also measured for dentin; the values obtained being presented in Figure 3B. For healthy dentin, the values obtained correspond to a fairly smooth and very compact surface at a fine microstructural level. As the treatment time progresses, changes in the morphology of HAP crystallites occur, which change the surface topography. Statistical significance (*p* < 0.05) was reached for Ra at 12 h and 48 h, respectively.

Untreated healthy dentin exhibits at the nanostructure level a compact structure of HAP nanoparticles (Figure 3C.a). They have rounded shapes and diameters mainly of 40 nm and are very well trapped in the collagen structure, resulting in a very compact composite material. The fact that the nanostructure of collagen fibers was not seen attests to the high degree of mineralization of the healthy dentin.

Side effects of I-131 treatment act in the same way on HAP crystallites in dentin as in those in enamel. It was noticed that their morpho-dimensional changes occurred only after 12 h of treatment when the crystallites became slightly oval. This process occurred due to the collagen that binds the nanoparticles to HAP keeping them in a dense tissue. The only signs of alteration at the nanostructure level was a relative increase in the distance between HAP crystallites, which could lead to increases in the roughness value. After 24 h of treatment, significant nanostructure changes occurred with increases in the diameter of the crystallites and with a clearer observation of the collagen strands, which would correlate with a local demineralization (Figure 3C.e). Alteration of the dentin nanostructure progresses in proportion to the increase in treatment time. The nanostructure of the dentin after 192 h, shows radically changed, compared to the untreated sample (Figure 3C.h). It was observed that the smallest nano-formations have a diameter of about 80 nm, which means that the HAP nanoparticles in the dentin have undergone changes similar to those in enamel. The resulting morphology can be correlated with the appearance of a partially demineralized dentin under acidic conditions.

The roughness’ Ra (at 6 h, 12 h) and Rq (at 12 h) showed statistical significance (*p* < 0.05) at nanostructure level (Figure 3D), which correlates with the observed morphological changes. The variation of the diameter of the HAP crystallites in the dentin (Figure 3E) showed a logarithmic appearance correlating with the behavior of those in the enamel. More interesting is the 40 nm level observed between 0 and 12 h of treatment which showed the higher resistance of the dentin to the deformation tendency under the action of gamma radiation. One explanation would be the positioning of the dentin at the inside of the tooth, not having direct contact with the incident waves, these being relatively attenuated by the enamel. On the other hand, the resistance to shape change may be due to the presence of collagen that binds nanoparticles to HAP.

## 4. Discussion

Iodine-131 is an unstable radioisotope of iodine that is used for the treatment of benign and malignant thyroid diseases, being absorbed by the follicular cells via the sodium/iodine symporter. Radioiodine I-131 decays with a half-life of 8.02 days with beta minus decay and gamma emission [3,19].

The primary emissions of I-131 decay are electrons with a maximal energy of 606 keV (89% abundance, others 248–807 keV) and 364 keV gamma rays (81% abundance, others 723 keV). The electrons, due to their high mean energy (190 keV, with typical beta-decay spectra present) have a tissue penetration of 0.6–2 mm [3,19].

Unevenness on the surface of healthy enamel can have different causes such as, on the one hand, the mechanical effect of mastication, or on the other hand, the mechanical effect of sample preparation. This is due more to the local topographic characteristics of the enamel than to the treatment itself. In this situation, except for certain extreme values in the graph, it can be seen an increasing trend of roughness that could be correlated with the effect of treatment. The roughness measured at the nanostructure level is more adequate to characterize the treatment-induced effects due to the possibility of avoiding areas with local topographic influences.

The most plausible explanation is the conjunction of two major effects mentioned in the literature. Firstly, is the alteration of the crystal lattice of HAP prisms due to the penetration of gamma radiation in enamel [20]. This can lead to a change in the volume of the elementary cells that manifests itself with a relative increase in diameter (e.g., from 40 to 45 or even 60 nm). This intracrystalline disorder appearing explains the appearance of a spacing between the prisms of HAP as if the structure of the enamel were to crack. The second effect mentioned in literature as a consequence of exposure to gamma radiation of enamel is demineralization [11,21]. This is manifested by loss of HAP prisms from the enamel surface at the nanostructure level (something similar to what it was began to be observed in Figure 2C.c after 6 h of treatment). If the dislocated prisms can leave the surface of the enamel and cavities appear which are likely to increase the surface roughness, in the inner mass of the enamel it is difficult for the dislocated prisms to be removed. Therefore, with the increase of the treatment time, the nanostructure dislocations progress so that from the compact structure of the healthy enamel, rocky structures are formed which in their interior have a cohesion close to the healthy enamel (several HAP prisms that have remained well welded together).

The overlap between the two effects described above is the driving force for altering the structure of tooth enamel due to gamma radiation treatment. The mechanism of alteration can be tested by mechanical tests of compressive strength or Vickers hardness. Nanostructure dislocation must significantly reduce the compressive strength and Vickers hardness, respectively. The Vickers imprint is expected to be much larger on the treated enamel for 192 h than on the untreated one.

Some data from the literature indicate that both, enamel and dentin, in addition to local demineralization show melting zones alternating with crack areas highlighted by SEM microscopy [22,23]. The areas destroyed by melting observed by them have a circular appearance with diameters around 10–50 μm and the cracks have lengths of about 30 μm with a width of about 1 μm (their depth can be estimated as average, sometimes superficial). These destructions mentioned in the literature are characteristic of the coarse and medium microstructure.

At the level of the fine microstructure and nanostructure of the samples investigated by our research team, there is no evidence of melting of the enamel or dentin due to radioiodine. However, we can retain another hypothesis that would explain the increase in the diameter of HAP crystallites at long treatment times. This would be that under certain conditions, due to radiation, the diffusion appears between the connected nanoparticles forming in the first phase clusters and then larger compact formations.

## 5. Conclusions

The AFM investigation of human enamel and dentin samples subjected to I-131 treatment in DTC shows that the enamel is the first to be affected: the nanostructure starts to change up to 6 h of treatment, and the fine microstructure after 6 h of treatment. Dentin begins to be affected after 12 h of treatment.

The degradation of enamel and dentin starts from the alteration of the crystalline network of HAP prisms, which is manifested by increases in diameter and the formation of a network of submicron cracks. They alter the compactness of the enamel and then the dentin, transforming them from compact materials into an agglomeration of rocky submicron structures (diameter 200–500 nm) still well welded together but which can easily generate cracking at the intercrystalline level.
